# Endemic zoonoses in the tropics: a public health problem hiding in plain sight

**DOI:** 10.1136/vr.h798

**Published:** 2015-02-28

**Authors:** Jo E. B. Halliday, Kathryn J. Allan, Divine Ekwem, Sarah Cleaveland, Rudovick R. Kazwala, John A. Crump

**Affiliations:** 1 College of Medical, Veterinary and Life Sciences, University of Glasgow, Glasgow G12 8QQ, UK; 2 Faculty of Veterinary Medicine, Sokoine University of Agriculture, P. O. Box 3015, Chuo Kikuu, Morogoro, Tanzania; 3 Centre for International Health, Dunedin School of Medicine, University of Otago, PO Box 56, Dunedin 9054, New Zealand

## Abstract

Zoonotic diseases are a significant burden on animal and human health, particularly in developing countries. Despite recognition of this fact, endemic zoonoses often remain undiagnosed in people, instead being mistaken for febrile diseases such as malaria. Here, as part of *Veterinary Record*'s ongoing series of articles on One Health, a multidisciplinary team of researchers from Scotland, Tanzania and New Zealand argues that a One Health approach is needed to effectively combat these diseases

THE resurgence of interest in One Health over the past decade has been fuelled by global concerns relating to zoonotic diseases with pandemic potential, such as highly pathogenic avian influenza A H5N1, severe acute respiratory syndrome coronavirus (SARS-CoV) and Ebola. Early advocates of One Health such as Calvin Schwabe became aware of the importance of integrating veterinary and medical approaches through work on endemic diseases of people and livestock (Schwabe 1984).[Fig VETRECH798F1]

**Figure VETRECH798F1:**
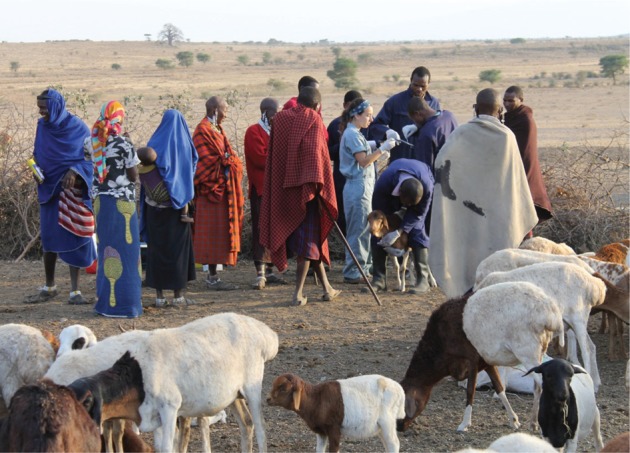
Study team visiting a pastoral community in Tanzania. The collection of data on the presence and effects of zoonoses in linked animal and human populations is crucial to understanding the epidemiology and overall impacts of these diseases

Today, endemic zoonoses continue to inflict an enormous disease burden, particularly across tropical regions. Endemic zoonoses affect human health and wellbeing directly as common causes of human disease, and indirectly through impacts on livelihoods and food security as a result of livestock production losses. Despite these multiple impacts, endemic zoonoses are still rarely recognised and are poorly understood. Their widespread mismanagement contributes to a vicious cycle of ill-health and poverty. Here, we review the factors that contribute to the ‘invisibility’ of endemic zoonoses as a global health problem with a focus on Africa, and highlight the crucial importance and value of One Health approaches to effectively tackle these diseases.

‘Even well-recognised zoonotic diseases with distinctive clinical signs may be misdiagnosed as malaria’

## Non-specific disease syndromes

Endemic zoonoses such as brucellosis (*Brucella* species), Q fever (*Coxiella burnetii*), leptospirosis (*Leptospira* species), rickettsioses (*Rickettsia* species), bartonellosis (*Bartonella* species), plague (*Yersinia pestis*), Rift Valley fever and Chikungunya (both caused by arboviruses), and many others, pose considerable challenges for clinicians in both human and animal health. They frequently present with general symptoms that are shared with a wide range of infectious diseases common in the tropics, and are hard to identify or differentiate clinically. As a consequence, the true burden of endemic zoonoses is largely underappreciated and awareness among clinicians and policymakers remains limited.

In humans, non-specific symptoms such as fever, headache, fatigue, and joint or muscle aches are commonly associated with many endemic zoonoses. These symptoms also occur with common non-zoonotic diseases, such as malaria and typhoid fever, which are likely to be considered more readily by clinicians (Crump 2012, 2014). Considerable social influences, such as training context, the influence of peers, and pressure to meet patient expectations, can also contribute to the overdiagnosis of diseases such as malaria, and thus to the relative underdiagnosis of other diseases including many zoonoses (Chandler and others 2008). Even well-recognised zoonotic diseases with distinctive clinical signs may be misdiagnosed as malaria. For example, in a study of childhood encephalitis in a malaria-endemic region of Malawi, rabies was confirmed as the cause of 10.5 per cent of fatal cases of encephalitis. Several of these cases were originally attributed to cerebral malaria, with clinical manifestations indistinguishable from those of cerebral malaria, but with subsequent histological examination showing no evidence of sequestration of parasitised erythrocytes in cerebral tissues (a hallmark of cerebral malaria) (Mallewa and others 2007).

More specific symptoms may occur with some zoonotic diseases, but these lack sensitivity or specificity, so cannot be relied upon for a clinical diagnosis. For example, hepatomegaly and splenomegaly are often reported in cases of human brucellosis (World Health Organization [WHO] and others 2006), but vary in the degree to which they are observed within and between different populations (Bouley and others 2012, Dean and others 2012a). In northern Tanzania, 18.8 per cent of patients with a confirmed diagnosis of acute brucellosis also had hepato- or splenomegaly on physical examination (Bouley and others 2012). Similarly, pneumonia is often considered one of the main presentations of Q fever in humans; however, in a hospital-based study in Taiwan, only 13.5 per cent of confirmed cases of acute Q fever presented with respiratory symptoms (Lai and others 2014).

Epilepsy is one of the most common neurological conditions in Africa, estimated to affect 4.4 million people and having significant physical, economic and social consequences (Paul and others 2012). Neurocysticercosis, caused by the tapeworm *Taenia solium*, is increasingly recognised as a major cause of epilepsy, with a meta-analysis of studies in Latin America, India and sub-Saharan Africa identifying neurocysticercosis as the cause of 30 per cent cases of epilepsy (Ndimubanzi and others 2010). Other endemic parasitic zoonoses that contribute to neurological syndromes in the tropics include *Trypanosoma brucei rhodesiense*, the zoonotic cause of human African sleeping sickness; and toxoplasmosis, which is the leading cause of central nervous system (CNS) disease in HIV-infected patients. A wide range of zoonotic parasitic diseases, including echinococcosis and trichinosis, as well as non-parasitic zoonoses, such as leptospirosis and borreliosis, also have the potential to cause a range of CNS signs, and may contribute to the burden of neurological disease in endemic areas.

‘Even where clinical signs of disease are seen . . . the level of disease recognition and reporting is likely to be several fold lower in livestock than humans, ensuring that animal healthcare providers often have even less observational data to inform diagnoses than their medical colleagues’

The challenge of non-specific presentation of many zoonoses also applies to the diagnosis of animal infection. For some zoonoses, for example *Escherichia coli* O157 in livestock and *T brucei rhodesiense* in wildlife, zoonotic infections often cause no apparent clinical signs in the animal host. Even where clinical signs of disease are seen (for example, with Q fever, brucellosis and leptospirosis), the level of disease recognition and reporting is likely to be several fold lower in livestock than in humans, ensuring that animal healthcare providers often have even less observational data to inform diagnoses than their medical colleagues.[Fig VETRECH798F2]

**Figure VETRECH798F2:**
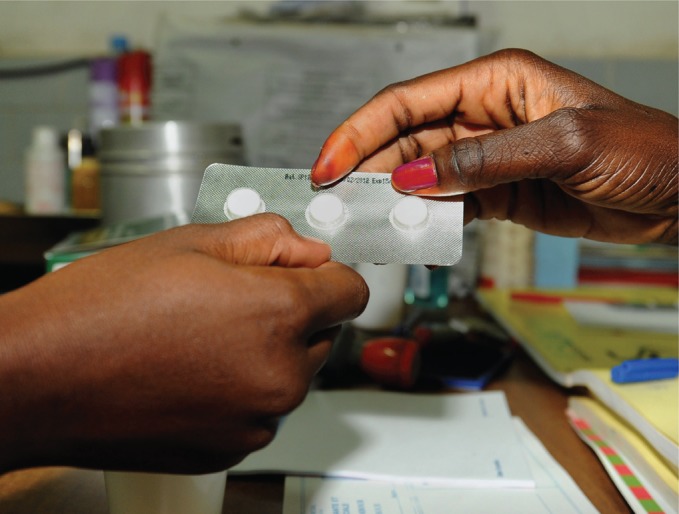
A doctor hands over malaria medication at a hospital in Senegal. Studies have shown that, in tropical regions, zoonotic diseases in people are often misdiagnosed as malaria as they have similar symptoms

Abortions are often one of the most readily recognisable signs of infectious illness in livestock and can have severe impacts on individual animal and herd productivity. Data concerning the incidence of livestock abortions and other productivity measures are frequently lacking in livestock-dependent settings. However, a study in northern Tanzania reported abortion events in 19 per cent of cattle herds, and 33 per cent of sheep/goat flocks, with 12.9 per cent of female domestic ruminants having a history of at least one abortion (Shirima 2005). Infection with several of the priority zoonoses of the World Organisation for Animal Health (OIE), including *Brucella*, *Leptospira* and *Streptococcus* species, *Campylobacter*, *Chlamydia*, *Ehrlichia*, *Anaplasma*, *Borrelia burgdorferi* and *C burnetii* can cause abortion in livestock species and other animals. The fact that so many zoonoses affecting people in the tropics also cause abortion in livestock suggests that there is likely to be great value in One Health approaches that link aetiological and epidemiological studies of livestock abortion with research on common human health syndromes.

## Diagnostic capacity

In many developing countries, a lack of laboratory diagnostic capacity adds to the challenges that clinicians face in establishing a diagnosis for zoonotic causes of human illness. Even with thorough history taking and careful evaluation of clinical signs, the number of differential diagnoses for common disease syndromes can be large and capacity to conduct reliable diagnostic tests for the possible aetiologies is often limited. The lack of neurological imaging facilities, such as computerised tomographic scans, contributes substantially to a lack of information on the causes of neurological syndromes.[Fig VETRECH798F3]

**Figure VETRECH798F3:**
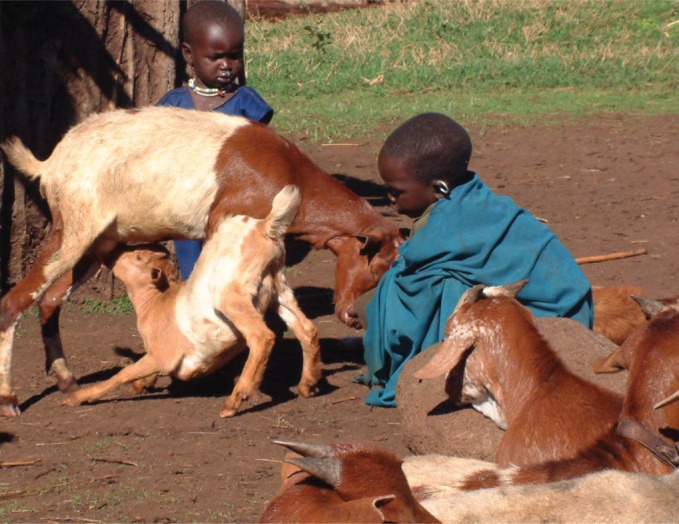
Goats and children from a household in Tanzania in which both people and livestock had been affected by brucellosis

‘The fact that so many zoonoses affecting people in the tropics also cause abortion in livestock suggests that there is likely to be great value in One Health approaches that link aetiological and epidemiological studies of livestock abortion with research on common human health syndromes’

There are few laboratories in Africa with the capacity to perform direct pathogen isolation or detection in acutely ill patients by blood culture or by molecular diagnostic assays such as nucleic acid amplification by PCR (Petti and others 2006). Culture and isolation of many zoonotic pathogens can be hazardous and laboratory-acquired infections pose a real risk for laboratory personnel. Attempts to culture Hazard Group 3 pathogens such as *Brucella*, *Coxiella* and *Mycobacterium* species should only be made in diagnostic laboratories with appropriate containment facilities, which are few and far between in low-income countries.

Serological diagnostic tests are more widely available and safer to conduct; however, many rely on demonstrating rising antibody titres for definitive diagnoses. As this is measured in paired acute and convalescent samples collected over a two- to six-week period, diagnoses based on seroconversion are only made weeks after initial patient presentation and cannot inform the clinical management of acutely ill patients.

High levels of ‘background’ exposure in some populations can complicate the interpretation of serological data for endemic pathogens (Levett 2001). Furthermore, many existing serological tests are hampered by inadequate test performance, including poor specificity, reproducibility and reliability. For example, a study of brucellosis in Kenya demonstrated a poor agreement between serological test results from four rural health facilities when compared with results from the central veterinary laboratory (Maichomo and others 1998).

Diagnosis of zoonotic infections in animals is also difficult in resource-limited settings. Historically, epidemiological studies have relied mainly on serological surveys that demonstrate the extent of exposure in different animal populations. However, data gathered through serosurveys cannot inform understanding of pathogen shedding dynamics, which is critical for understanding zoonotic transmission risks. In many cases, serological assays also lack the specificity to differentiate between pathogen species or strain types. Serological tests cannot distinguish between infections with *Brucella abortus* or *Brucella melitensis*, for example, and there is considerable cross-reactivity between different *Leptospira* serovars. This lack of specificity has important implications for the identification of sources of infection and for the development of vaccination strategies. The use of culture and molecular methods for pathogen detection in animals has also been limited by many of the infrastructure and logistical restrictions that affect human disease diagnostics. In combination with a lack of research prioritisation, these factors play an important role in perpetuating low levels of knowledge and awareness of zoonotic pathogens circulating in livestock, domestic animals and wildlife populations.

## Delays in case presentation

Delayed healthcare-seeking behaviour of patients, coupled with the chronic nature of many zoonotic conditions, can further compound challenges in acute disease diagnosis as detection of pathogens can be difficult at later stages of infection. A study of healthcare-seeking behaviour among patients diagnosed with brucellosis in northern Tanzania revealed that just 22 per cent of cases sought care at a hospital within the first month after the onset of their symptoms (Kunda and others 2007). The sensitivity of blood culture for diagnosis of brucellosis is low even in the acute phase of disease and falls even further in the diagnosis of longstanding disease (WHO and others 2006). Similarly, in patients with leptospirosis, leptospires can only be detected in the blood of an infected person within the first week of clinical illness. Selection of appropriate diagnostic specimens is dependent on timing and requires careful history taking with regards to the timing of onset of clinical symptoms (Haake and Levett 2015). Isolation rates from blood culture can be very low even if patients are sampled during the acute febrile stages of illness (Levett 2001). In the absence of an acute phase sample, serological diagnosis is complex and often inconclusive for most endemic zoonoses.

‘In a prospective cohort study involving 870 febrile patients in northern Tanzania, malaria was the clinical diagnosis in the majority of cases (60.7 per cent), but was the actual cause of fever in very few (1.6 per cent)’

## Neglected diseases in neglected populations

Many zoonotic diseases that are overlooked in endemic settings are not universally neglected. Brucellosis, for example, has been well-studied and eradicated in several countries with highly developed commercial livestock sectors (Dean and others 2012b). Endemic zoonoses remain widely neglected in many low-income settings because their impact is borne largely by impoverished and marginalised communities (Molyneux and others 2011). They disproportionately affect people who are not only at high risk of pathogen exposure but also have little access to adequate primary healthcare (ILRI 2012).

Even when reliable point-of-care diagnostic tests become more widely available for clinical management of common illnesses, health facilities serving these communities are those least likely to be able to establish diagnostic capacity. Inequalities in access to healthcare facilities are also likely to be a major contributing factor to underdiagnosis and under-reporting of zoonotic disease in Africa and other tropical regions (Molyneux and others 2011, ILRI 2012). Consequently, zoonotic diseases of impoverished communities continue to be overlooked in global disease control priorities, and the cycle of neglect is perpetuated by the lack of reliable data on incidence and impact.

## Assessing the impacts

There are many steps in quantifying the overall impact of a given disease. Identification of individual cases is the first step in the process. Measures of disease incidence alone are only a first step towards prioritisation of investment in disease control to improve overall public health, as they do not encompass any information about the impacts of a disease on an individual or population.

In human health, a variety of measures have been developed to quantify the multiple impacts of disease, such as quality of life and economic impacts of disease. The Global Burden of Disease study of the WHO and many other organisations and studies use disability-adjusted-life-years (DALYs) to measure and compare the burden of a wide range of different diseases and enable rational prioritisation of investment in healthcare. DALYs combine years of life lost to premature mortality and years of life lost due to time lived in states of less than full health to measure the impact of illness on an individual (WHO 2015). However, DALYs do not incorporate any measure of other kinds of disease impact, such as the economic costs of illness for the individual or society (Grace and others 2012). Crucially for the evaluation of the impact of zoonoses, DALYs do not include any measure of the impact of zoonoses upon animal health either directly or through the costs of lost animal productivity. These may include, for example, the impact of reduced milk production on child health and nutrition; the economic impacts of reduced reproductive success or abortion in commercially valuable livestock species, and the secondary consequences of the loss of draught power for subsistence farmers.

While frameworks exist for economic evaluation of zoonoses (Narrod and others 2012), there are currently no standardised metrics equivalent to the DALY that integrate the multiple impacts of zoonoses on human and animal health. Consequently, the burden of these diseases is, at best, only partially quantified and their significance often underestimated, particularly when viewed alongside diseases that affect only human health (Grace and others 2012).

## One Health approaches

Given the considerable challenges in the diagnosis and management of endemic zoonotic diseases in Africa, interest has been growing in the benefits of adopting a more integrated One Health approach that involves both human and animal health sectors. The sections below outline the rationale, application and added value of these approaches. We also provide illustrations of the progress that has been made through adoption of these approaches and highlight some priorities for future work.

Several comprehensive aetiological studies using gold-standard diagnostics have now been conducted in Africa and Asia to better define the contribution of a broad spectrum of infectious diseases to common clinical syndromes such as febrile or neurological illness. These studies invariably reveal that zoonotic infections cause substantial proportions of human illness, and confirm high levels of misdiagnosis of zoonotic illnesses (Suttinont and others 2006, Gasem and others 2009, Manock and others 2009, Kendall and others 2010, Crump and others 2013).

For example, in a prospective cohort study involving 870 febrile patients in northern Tanzania (Crump and others 2013), malaria was the clinical diagnosis in the majority of cases (60.7 per cent), but was the actual cause of fever in very few (1.6 per cent). In fact, bacterial zoonoses, which were not initially considered by clinicians in any cases, were confirmed through reference laboratory diagnostic testing of paired acute and convalescent serum samples as a cause of disease in 26.2 per cent of all febrile admissions. These zoonoses included leptospirosis (8.8 per cent of all febrile admissions), spotted fever group rickettsioses (8.0 per cent), Q fever (5.0 per cent), brucellosis (3.5 per cent) and typhus group rickettsioses (0.4 per cent). In two sites (one rural, one urban) in southern Tanzania, leptospirosis, Q fever, toxoplasmosis and rickettsial infections were also identified as causes of febrile illness in people (D'Acremont and others 2014).[Fig VETRECH798F4]

**Figure VETRECH798F4:**
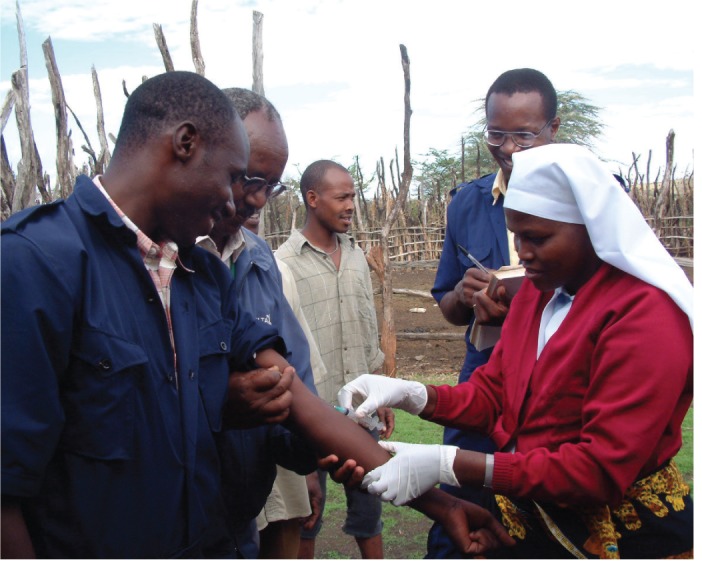
Blood sampling of Tanzanian livestock workers at risk of infection with brucellosis

Aetiological studies such as these help to raise awareness of the presence and importance of many zoonoses. Unfortunately, however, these studies typically employ diagnostic approaches that provide retrospective diagnoses only or tests that are not routinely available in many settings and cannot necessarily be extended to provide diagnostic solutions for primary healthcare providers.

‘Zoonotic diseases of impoverished communities continue to be overlooked in global disease control priorities, and the cycle of neglect is perpetuated by the lack of reliable data on incidence and impact’

## Intersectoral communication

Many of the same endemic zoonotic pathogens occur widely in both human and animal populations across Africa and other tropical regions, and there is substantial information available on distribution patterns, largely through prevalence surveys. This is demonstrated in systematic literature reviews of brucellosis (Dean and others 2012b, Rubach and others 2013), Q fever (Vanderburg and others 2014), leptospirosis (de Vries and others 2014, K. J. Allan, personal communication), cysticercosis (Zoli and others 2003, Winkler 2012, Assana and others 2013), *Toxoplasma gondii* infection (Hammond-Aryee and others 2014) and zoonoses overall (ILRI 2012). However, there is still a need for improved communication of information across sectors to inform diagnosis, clinical management and disease control strategies.

Many endemic zoonoses should be considered in the list of possible aetiologies for relevant human disease syndromes in most, if not all, parts of Africa. However, there is considerable local variation in prevalence and incidence in both human and animal populations globally, which suggests a need for greater understanding of local patterns of persistence and specific risk factors for animal and human infections in different environments. This understanding will be important for establishing evidence-based public health policy and in developing clinical algorithms for disease risk to guide clinicians in diagnostic test selection and patient management. For example, in East Africa the prevalence of brucellosis appears to be substantially higher in both human and animal populations living in pastoral communities than in smallholder farming areas (McDermott and Arimi 2002). Brucellosis should therefore be considered a more likely cause of febrile illness in patients living in high-risk communities. Exposure to *T solium* infection is associated with community-level pig ownership and lower levels of sanitation. Thus, neurocysticercosis should be considered as a differential diagnosis of epilepsy where pig-keeping is practised in the patient's home community (Assana and others 2013). Ongoing communication is required to enable the translation of findings in one sector into practice in the other.

For many endemic zoonoses, we still have a poor understanding of specific risk factors for both human and animal infection, and this lack of knowledge is likely to contribute substantially to under-recognition of clinical impacts of disease in animal and human populations. For example, exposure to *T gondii* occurs throughout Africa, with some indication of decreasing prevalence from north to south, and from west to east (Hammond-Aryee and others 2014). However, the specific risk factors underlying these geographic trends remain unknown. Q fever is similarly pervasive, and although risk factors for animal and human infection have been identified in some settings (for example, associations with camels in Chad [Schelling and others 2003], and owner's ethnic group in Cameroon [Mazeri and others 2013]), much remains unknown about epidemiological risk factors in Africa. *Leptospira* infections have also been detected in a wide range of animal hosts in Africa, including livestock and many wildlife species. However, marked variation in prevalence and strain type of infecting serovars exist and little is known about the factors that influence disease transmission in a wide range of agroecological settings. Additional linked analyses of these neglected zoonoses in human and animal populations are needed to: (i) synthesise the data that are currently available; and (ii) reveal key features of the epidemiology of these pathogens that can only be appreciated when the true multihost nature of that epidemiology is explicitly considered.

## Molecular epidemiology and new surveillance approaches

Increasingly, the rapid advancement of PCR-based nucleic acid detection methods and high throughput sequence analysis for strain identification is providing new opportunities to understand the multihost epidemiology of endemic zoonoses.

High-resolution genetic typing data allow the characterisation and comparison of pathogens present in humans and animal hosts, enabling identification of likely infection sources and transmission routes to people. To effectively use the disease control opportunities provided by these new diagnostic techniques (for example, identification of key intervention points), there is a need to develop novel surveillance approaches that enable targeted collection of diagnostic material suitable for application of these techniques. In most cases this will require responsive sampling of clinical cases, in which detection of the infecting pathogen is feasible, rather than cross-sectional sero-surveillance. There is great scope to develop syndromic approaches to disease detection and diagnostics in animal populations. For example, livestock abortions are often noticed by the farmer, and thus may provide a memorable event around which to base a livestock surveillance system for many key zoonotic pathogens.

Laboratory testing of diagnostic material from abortion events, including pathogen detection and molecular characterisation, has great potential for enhancing our understanding of the epidemiology of many infections in livestock populations. In the case of brucellosis, despite numerous seroprevalence surveys, very little is still known about the relative contribution of different *Brucella* species to the disease burden in both animal and human populations in East Africa. Being able to distinguish the role and risk factors for *B abortus* and *B melitensis* in different host species is clearly a fundamental and urgent requirement before effective control measures including animal vaccination can begin.

‘Interventions that may control zoonotic infection in animal populations or prevent disease transmission from animals to people may offer more effective and economically viable approaches to disease management than those focusing on the human population alone’

## Disease control at source vs fire-fighting

Perhaps the greatest added value to be gained through the adoption of One Health approaches is the opportunity to implement control programmes that reduce the multiple impacts of zoonoses in both human and animal populations. Interventions that may control zoonotic infection in animal populations or prevent disease transmission from animals to people may offer more effective and economically viable approaches to disease management than those focusing on the human population alone. This is particularly true where zoonotic infections have a detrimental effect on household livelihoods through the impacts of infection on livestock reproductive success and productivity.

Many effective interventions against zoonotic diseases are already available for livestock, with the potential to bring immediate benefits to both human and animal health. For example, effective livestock vaccines are available for the control of brucellosis, Q fever, leptospirosis, anthrax (*Bacillus anthracis*) and toxoplasmosis (*T gondii*). However, these have rarely been deployed in large-scale control programmes in low-resource settings, partly because the rationale for livestock vaccination tends to be argued only from the perspective of the livestock sector and in terms of private good to the livestock owner. Conversely, approaches to cost-effectiveness analysis of health interventions typically consider only the human health benefits, which represent only one component of societal benefits of zoonoses control. Incorporating public health benefits (for example, DALYs avoided), livestock production gains and livelihood benefits into these evaluations can substantially improve cost-effectiveness measures, and have provided a convincing rationale for advocating livestock vaccination against brucellosis in Mongolia (Roth and others 2003) and livestock interventions for control of trypanosomiasis (Shaw and others 2006).

Effective control of disease in animal host populations also has the potential to address issues of health equity through prevention of disease transmission to the whole community. By offering protection to those who are most at risk from infection as well as those least able to access appropriate healthcare when suffering from disease, a One Health approach offers more widespread and equitable benefits than relying on medical treatment at health facilities alone.

## Conclusion

The One Health paradigm encourages a holistic perspective. When considering endemic zoonoses, this breadth of perspective is essential to appreciate the full range of impacts of these diseases that are too often overlooked. The non-specific clinical presentation of many zoonoses, the complexity of diagnostics for these diseases and the relative lack of data on their clinical burdens all contribute to the under-recognition of their importance and thus to their ongoing neglect.

There is considerable potential for the application of molecular diagnostic approaches to greatly improve understanding of the distribution and fine-scale transmission processes of many endemic zoonoses. However, even with the widespread availability of cutting-edge diagnostic capacities, the diagnosis of many zoonoses will remain challenging and it is clear that improved front line diagnostic capacity alone cannot be the only measure taken to effectively tackle the multiple impacts of these diseases.

As well as efforts to build diagnostic capacity and improve the management of individual cases, there is a need to implement preventive and control measures that tackle these diseases in a much more fundamental way. This research should include studies into the practical implementation and evaluation of disease control programmes that reduce transmission in animal populations, control zoonotic diseases at their source and thus reduce their impacts on human health, animal health and livelihoods.
